# Lack of Thromboxane Synthase Prevents Hypertension and Fetal Growth Restriction after High Salt Treatment during Pregnancy

**DOI:** 10.1371/journal.pone.0151617

**Published:** 2016-03-14

**Authors:** Chen-Hsueh Pai, Ching-Tzu Yen, Chie-Pein Chen, I-Shing Yu, Shu-Wha Lin, Shu-Rung Lin

**Affiliations:** 1 Department of Clinical Laboratory Sciences and Medical Biotechnology, College of Medicine, National Taiwan University, Taipei, Taiwan; 2 Center of Genomic Medicine, National Taiwan University, Taipei, Taiwan; 3 Division of High Risk Pregnancy, Department of Obstetrics and Gynecology, Mackay Memorial Hospital, Taipei, Taiwan; 4 Laboratory Animal Center, College of Medicine, National Taiwan University, Taipei, Taiwan; 5 Department of Bioscience Technology, College of Science, Chung-Yuan Christian University, Taoyuan, Taiwan; 6 Center for Nanotechnology and Center for Biomedical Technology, Chung-Yuan Christian University, Taoyuan, Taiwan; Taipei Medicine University, TAIWAN

## Abstract

Preeclampsia (PE) is a potentially fatal pregnancy-related hypertensive disorder characterized by poor placenta development that can cause fetal growth restriction. PE-associated pathologies, including thrombosis, hypertension, and impaired placental development, may result from imbalances between thromboxane A_2_ (TXA_2_) and prostacyclin. Low-dose aspirin, which selectively inhibits TXA_2_ production, is used to prevent high-risk PE. However, the role of TXA_2_ in aspirin-mediated protective effects in women with PE is not understood fully. In this study, we examined the role of prostanoids in PE using human samples and an induced PE mouse model. We demonstrated that the administration of salted drinking water (2.7% NaCl) to wild-type mice resulted in elevated placental TXA_2_ synthase (TXAS) and plasma TXA_2_, but not prostacyclin, levels, which was also found in our clinical PE placenta samples. The high salt-treated wild-type pregnant mice had shown unchanged maternal body weight, hypertension (MAP increase 15 mmHg), and decreased pup weight (~50%) and size (~24%), but these adverse effects were ameliorated in TXAS knockout (KO) mice. Moreover, increased expression of interleukin-1β and downstream phosphorylated-p38-mitogen-activated protein kinase were concordant with apoptosis induction in the placentas of salt water-treated wild-type mice. These alterations were not observed in TXAS KO mice. Together, our data suggest that TXA_2_ depletion has anti-PE effects due to the prevention of hypertension and placental damage through downregulation of the interleukin-1β pathway.

## Introduction

Preeclampsia (PE) is a serious complication of pregnancy that is associated with high morbidity and mortality in affected mothers and children. Women with PE may have symptoms such as hypertension, proteinuria, renal insufficiency, hemolysis, reduced platelet count, and/or increased platelet activation. The present management of patients with PE depends on symptom severity, and selective drugs targeting mild to severe PE (e.g., methyldopa, hydralazine, magnesium sulfate) are available [1]. Nevertheless, the best treatment currently available for PE, particularly for patients diagnosed after gestational week 38 and those with maternal compromise or eclampsia after gestational week 20, is delivery of the infant and placenta. Consequently, PE contributes to the incidences of intrauterine fetal growth retardation (IUGR) and preterm birth.

Nearly 5–10% of women develop hypertension during pregnancy, and pregnancy-induced hypertension (PIH) is one of the most prevalent risk factors for PE [2]. Hypertensive conditions are multifactorial; factors associated closely with PIH and PE are inadequate implantation and defective cytotrophoblastic invasion of the maternal spiral arteries, resulting in poor placentation and placental dysfunction [3, 4]. Defective placentation may lead to focal regions of hypoxia, which, in turn, are thought to alter the production of growth factors, cytokines [5], lipid peroxides [6], and prostaglandins by placental trophoblasts [7]. For example, elevated placental levels of inflammatory cytokines, such as tumor necrosis factor-α, interleukin (IL)-1α, IL-1β, and IL-6, are generally considered to be unfavorable to pregnancy [8]. Moreover, clinical studies have shown changes in the levels of cytokines and prostaglandins in women with PE [9, 10]. These observations have raised interest in understanding the effects of these cytokines on placentation and disease progression in relation to hypertension and PE [11].

The balance between thromboxane A_2_ (TXA_2_) and prostaglandin I_2_ (PGI_2_) is altered in women with PE, and high levels of TXA_2_ metabolite have been detected in circulation in these patients [12, 13]. TXA_2_ and PGI_2_, derivatives of arachidonic acid, are functional antagonists. TXA_2_ stimulates platelet activation and aggregation, vessel constriction, and proliferation and mitogenesis of vascular smooth muscle cells, whereas PGI_2_ is an inhibitor of platelet aggregation and a vasodilator [2]. During placental ischemia/hypoxia caused by aberrant implantation, PGI_2_ synthesis may be downregulated and TXA_2_ synthesis upregulated. Moreover, the release of TXA_2_ from basal trophoblasts appears to be increased in placentas affected by PE [14]. High levels of TXA_2_ have been suggested to play a role in placental cell apoptosis, which may contribute to hypertension [15]. Abnormally elevated TXA_2_ levels are also known to induce thrombosis [16]. Thus, elevated TXA_2_ levels may explain the major clinical symptoms of PIH and PE, such as hypertension, platelet aggregation, and reduced uteroplacental blood flow [17, 18].

Low-dose aspirin has been used for many years to prevent PE. This treatment has led to 10% reductions in the prevalence of PE and delivery before 34 gestational weeks [19]. At low dosages, aspirin selectively blocks TXA_2_ synthesis, tipping the balance between TXA_2_ and PGI_2_ in favor of PGI_2_, which may improve uteroplacental circulation [20]. However, aspirin usage during pregnancy has led to controversial outcomes in clinical patients and animal models [11]. Low-dose aspirin has been shown to reduce the risks of PE development and TXA_2_-mediated damage [21], but it is useless in women at high risk of PE and can have side effects, such as postpartum bleeding and epigastric pain, in pregnant women [22]. Moreover, TXA_2_ did not contribute to hypertension or renal vasoconstriction in reduction-in-uteroplacental-perfusion-pressure rats [23], and did not decrease effects on the uterine artery in pregnant guinea pigs [24]. In contrast, other studies have shown that TXA_2_ analogs cause hypertension in pregnant animals [25, 26] and that ozagrel, a TXA_2_ modulator, can reduce PIH and proteinuria in PE [27]. Thus, the role of TXA_2_ in PE remains unclear.

Studies in rats have shown that high salt treatment in late pregnancy induces maternal hypertension and renal dysfunction [28, 29] and may have deleterious effects on the placenta. Placental defects may affect fetal development due to insufficient nutrient supply [30]. We generated TXA_2_ synthase (TXAS)-deleted mice and demonstrated that TXAS is not essential for embryogenesis, reproduction, growth, thrombopoiesis, or lymphocyte differentiation [31]. TXAS deletion causes a mild hemostatic defect, but protects mice against arachidonate-induced shock and death due to systemic platelet thrombi [31]. To investigate whether TXAS blockade may provide an alternative strategy for PIH and/or PE prevention, we used TXAS-deleted mice treated with high salt as a model in the present study.

## Materials and Methods

### Tissues

With approval of the Institutional Review Board of Mackay Memorial Hospital, Taipei, Taiwan, placental tissues were obtained from spontaneous preterm births, normal-term births, and patients with PE (33–38 weeks of gestation) following Cesarean delivery in the absence of signs and symptoms of chorioamnionitis. PE was defined as gestational blood pressure elevation with proteinuria, usually occurring after 20 weeks of gestation, according to the American College of Obstetricians and Gynecologists guidelines [32].

### Animals

Male (n = 10) and female mice (n = 40) (aged 8–16 weeks) were used in this study. We generated TXAS KO mice and bred them for 10 generations on a BALB/c background [31]. Briefly, we deleted from the TXAS gene intron 9 and replaced it with an HPRT cassette to terminate TXAS transcription. These mice were viable and fertile. All mice were maintained in the Laboratory Animal Center of the Department of Bioscience Technology of Chung Yuan Christian University (CYCU). All animal experiments were approved by the Animal Welfare Board of CYCU and performed according to its guidelines. The protocol was approved by the Institutional Animal Care and Use Committee of CYCU.

### Treatment protocol and sample collection

The addition of a high salt solution to drinking water has been established to induce PE symptoms in rats [28, 29]. In this study, we treated mice with a 2.7% NaCl solution using the protocol shown in S1 Fig. Briefly, pregnant mice were given dH_2_O (control group) or 2.7% NaCl solution (treatment group) in drinking water on gestational days 12–18. After parturition, mice that had given birth were anesthetized by 2.5% tribromoethanol (250 mg/Kg) immediately and plasma was collected. Renal and placental tissues were collected rapidly and stored in formalin or snap frozen at -80°C.

### Physiological measurements and analysis

The blood pressure of pregnant mice was measured using the indirect tail-cuff method (Softron BP-98A tail blood pressure system; Japan). All mice were trained at D10. Body weight and foot size were recorded daily from gestational day 10. Urine samples were collected on gestational days 11, 14, and 18. Urinary protein and creatinine (CRE) levels were determined with assay kits (Bio-Rad, USA and BioAssay Systems, USA, respectively). Placental weight, pup weight, and pup nose—rump length were measured immediately after birth.

### Plasma collection and molecular detection

Blood was collected by cardiac puncture into heparin tubes and centrifuged for 15 min at 1,500 × g and 4°C to collect plasma. TXA_2_ and PGI_2_ concentrations were measured by enzyme immunoassays (catalog nos. 501020 and 515211; Cayman, USA). Blood urea nitrogen (BUN) and CRE concentrations were determined using assay kits (catalog nos. DIUR-500 and DICT-500; BioAssay Systems). Mouse soluble Flt-1 (sFlt-1) levels were measured by ELISA (catalog nos. DY471 and DY1320; R&D systems, USA). Electrolytes were detected with a TBA-120FR automated clinical analyzer (Toshiba, Japan). All assays were performed according to the manufacturers’ instructions.

### RNA preparation and mRNA expression

Total RNA was extracted from placental tissues using a TRIzol kit (Invitrogen, USA) and used for complementary DNA synthesis, as described previously [31, 33]. Human TXAS, human GAPDH (internal control for human gene), mouse Tbxas1, and mouse Gapdh (internal control for mouse gene) transcripts were detected by quantitative reverse-transcription PCR (qRT-PCR) using TaqMan gene expression assays (Hs01022706_m1, Hs02758991_g1, Mm00495553_m1, and Mm99999915_g1; Applied Biosystems, USA) according to the manufacturer’s instructions. Real-time qPCR was performed using a standard protocol on an ABI-7300 device (Applied Biosystems). Mouse Il1b, Il6, and gapdh (reference) expression was quantified by SYBR Green qRT-PCR using previously reported primers [34, 35]. The primer sequences were: IL-1β forward 5'-CAGGCAGGCAGTATCACTCA-3', reverse 5'-ATGAGTCACAGAGGATGGGC-3' and GAPDH forward 5'-CTGGAGAAACCTGCCAAGTA-3', reverse 5'-AAGAGTGGGAGTTGCTGTTG-3'. All samples were run in triplicate. The threshold cycle was defined as the fractional cycle number at which fluorescence passed the fixed threshold. Relative quantification of target gene expression was performed using the comparative CT method.

### Western blot

Total tissue protein (25 μg) was separated by SDS-PAGE (10% gel) and transferred to an Immobilon polyvinylidene difluoride membrane (Pall, USA).[36] Immunoblot analysis was conducted using antibodies against TXAS (catalog no. 160715; Cayman) and GAPDH (catalog no. GT239; GeneTex, Taiwan). The proteins were visualized using DuPont Western Blot Chemiluminescence Reagent (NEN Research, USA).

### Histological analysis, immunohistochemistry, and TUNEL assay

Placentas and kidneys were collected and weighed. The tissues were fixed in 10% buffered formalin for 24 h, embedded in paraffin, cut into 5–7-μm sections, and stored at 4°C in the dark. For histological analysis, the sections were dewaxed before staining with hematoxylin and eosin [31]. For immunohistochemical analysis, the dewaxed tissue sections were incubated with a rabbit polyclonal anti-TXAS (1:100 dilution, catalog no. ab39362; Abcam, USA) or anti—IL-1β (1:200 dilution, catalog no. ab9722; Abcam) antibody, or a rabbit monoclonal anti—phospho-p38–mitogen-activated protein kinase (MAPK) antibody (1:200 dilution, catalog no. 4511; Cell Signaling, USA) at 4°C overnight. After washing with phosphate-buffered saline containing 0.5% Tween 20, primary antibodies were detected using Super SensitiveTM immunohistochemical detection systems (Biogenex, USA) according to the manufacturer’s instructions. Hematoxylin counterstaining was used. For apoptotic cell detection, the sections were stained using TUNEL assay kits (S7110; Millipore). All control tissues were stained appropriately.

### Statistical analysis

Statistical analysis was performed by using Mann-Whitney test of Prism software (GraphPad, California, USA). Data are expressed as mean with SEs. A value of p<0.05 was considered significant.

## Results

### Increased sodium and chloride levels in plasma after high salt treatment

Plasma sodium and chloride levels were higher in high salt-treated WT and KO mice than in regular water—treated control groups (both P<0.05; Table 1), indicating that the experimental treatment was successful. Other parameters, such as potassium, total calcium, magnesium, serum CRE, and BUN levels, remained unchanged after high salt treatment in WT and KO mice. No mouse in the WT or KO group developed proteinuria or edema in the lower extremity (as measured by foot size), and sFlt-1 levels remained unchanged in all experimental groups (S2 Fig).

**Table 1:** Effects of 2.7% NaCl supplement on Plasma Electrolytes, Creatinine, and BUN.

	WT		KO	
	dH_2_O	NaCl	dH_2_O	NaCl
Sodium, mmol/l	158.5±5.54	169.5±7.7 *	160.17±7.65	172.85±7.9 *
Potassium, mmol/L	5.83±0.51	5.83±0.55	6.75±1.01	5.91±0.56
Chloride, mmol/L	130.67±7.89	142.75±9.74 *	129.83±6.94	141.43±10.41 *
Total calcium, mmol/L	1.34±0.39	1.23±0.18	1.55±0.18	1.54±0.39
Magnesium, mmol/L	0.59±0.18	0.54±0.04	0.62±0.12	0.61±0.13
Creatinine, mg/dL	0.21±0.12	0.16±0.1	0.32±0.16	0.28±0.14
Blood urea nitrogen, mg/dL	27.77±9.92	33.56±30.15	32.12±6.23	46.21±18.4

Values are means±SD. Each group number were 5.

* *P<0*.*05* compared with control groups

### Increased TXAS levels in placentas of pregnant women and mice with disease

**Fig 1:**
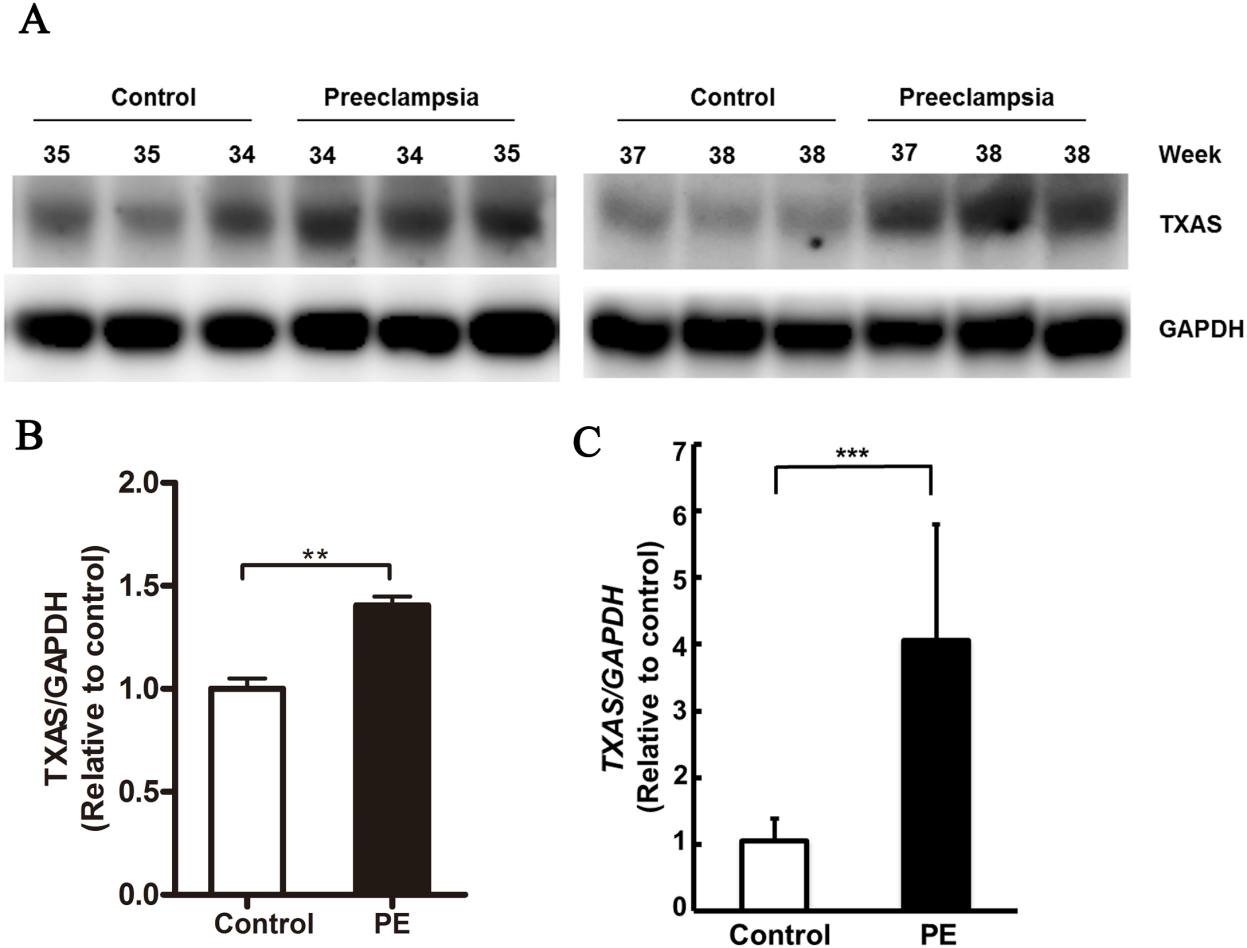
TXAS was upregulated in human PE placentas. (A) Western blotting of TXAS protein levels in placentas from women with PE and gestational age—matched controls. (B) TXAS protein was quantified by densitometric analysis (n = 6/group). (C) TXAS mRNA levels were determined by qRT-PCR (n = 15/group). Protein and mRNA expression levels of the reference gene GAPDH were used as internal controls for Western blotting and qRT-PCR, respectively. Expression relative to control patients is presented as mean±SEM. **P<0.01, ***P<0.001.

**Fig 2:**
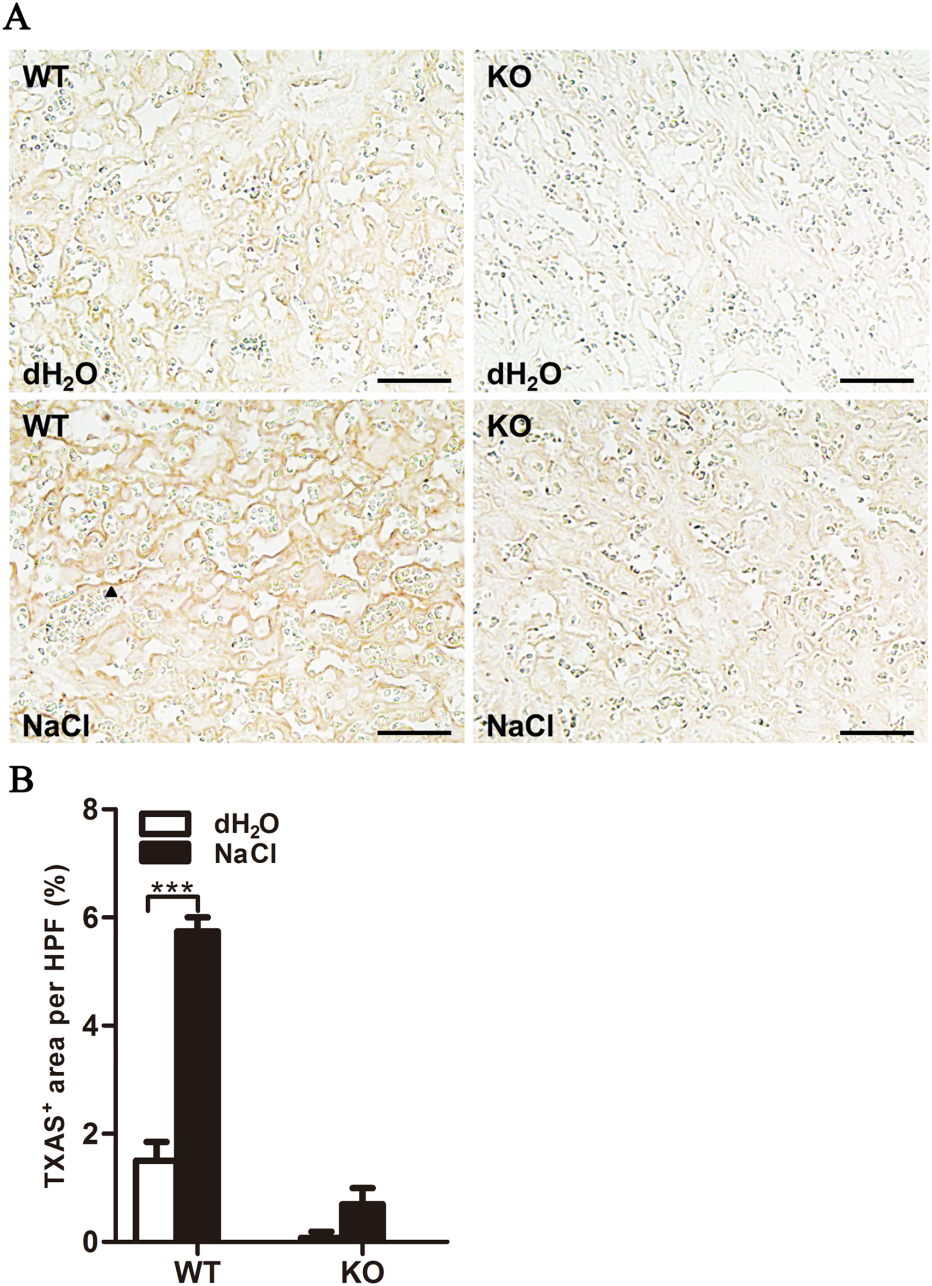
TXAS was upregulated in placentas after high salt treatment. (A) Immunohistochemical imaging of TXAS in placenta. Arrowhead shows a positive signal. Scale bar = 50 μm. (B) Quantification of TXAS-positive areas per high-power field (×200). n = 4/group. Data are presented as mean±SEM. ***P<0.001.

RNA and protein levels of TXAS were higher in the placentas of women with PE than in those of women without PE (P<0.001 and P<0.01, respectively; Fig 1). TXAS levels were also increased in pregnant WT mice compared with KO mice (P<0.001; Fig 2).

### High salt treatment induced PE-like phenotypes in mice

**Fig 3:**
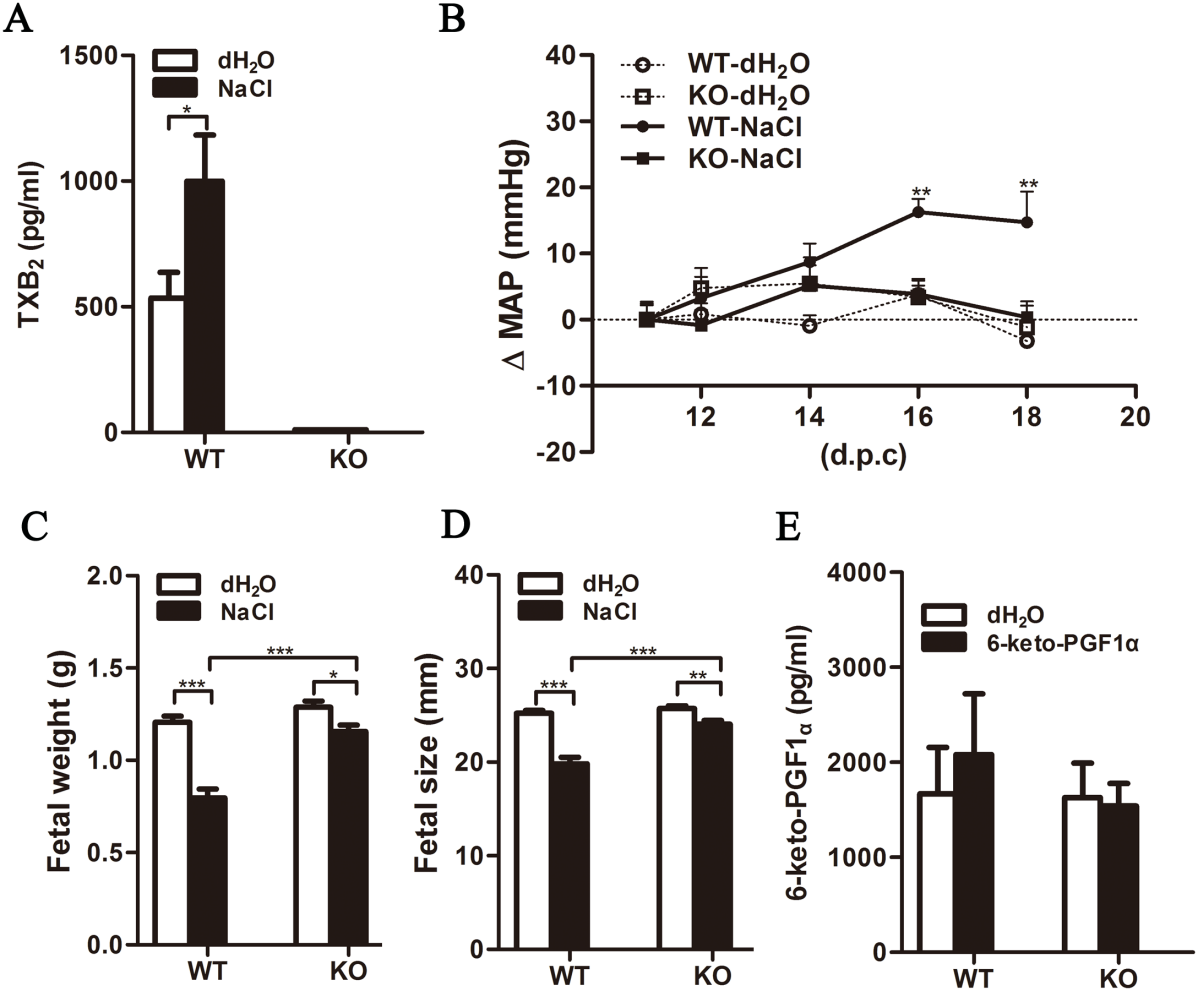
High salt treatment induced higher plasma TXA_2_ level and blood pressure, and affected fetal growth in pregnant WT mice. Plasma TXB_2_, a TXA_2_ metabolite (A), and 6-keto-PGF1α (E) levels were detected by EIA (n = 10/group). (B) MAP was measured from gestational days 11–18 and normalized to corresponding baseline pressure (n = 10/group). Arrow marks the beginning of high salt treatment. Pup weight (C) and size (D) were measured immediately after delivery. Data are presented as mean±SEM. *P<0.05, **P<0.01, ***P<0.001.

High salt—treated pregnant WT mice exhibited significantly increased levels of TXB_2_ (a metabolite of TXA_2_), as found in women with PE (P<0.05; Fig 3A). They also developed specific PE phenotypes, including high blood pressure and IUGR. In contrast, high salt-treated TXAS KO pregnant mice had undetectable TXB_2_ levels due to the loss of the TXAS gene, and had less severe phenotypes. High salt treatment lead to the gradual development of high blood pressure (deltaMAP = 15 mmHg, baseline MAP = 76 mmHg) in pregnant WT, but not KO, mice (Fig 3B and S2A and S2B Fig). The weight and size of pups born to high salt-treated WT mice were reduced by nearly 50% and 24%, respectively, whereas KO pups were only mildly affected (Fig 3C and 3D). 6-keto-PGF1α (a metabolite of prostacyclin) levels remained unchanged and comparable in the WT and KO groups during high salt treatment (Fig 3E), suggesting that prostacyclin did not play a role in this experimental model.

**Fig 4:**
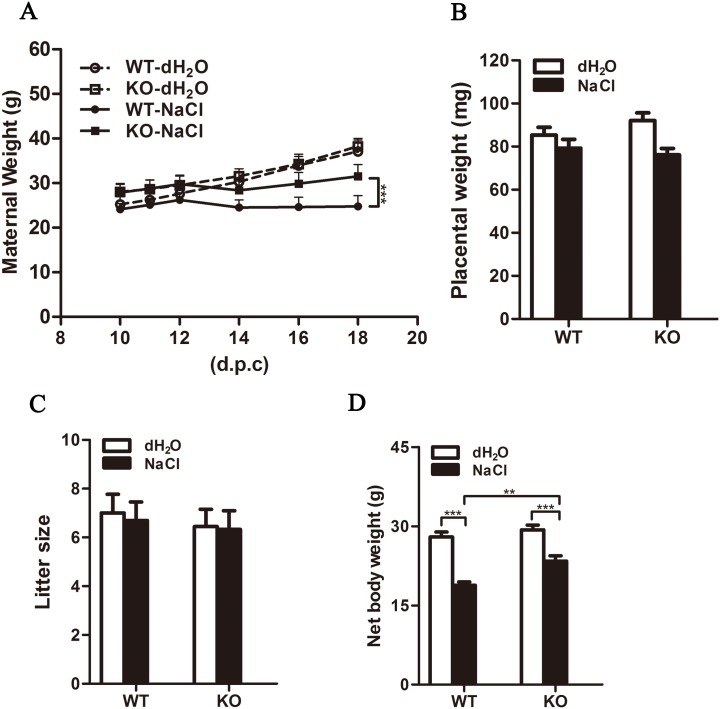
Effects of high salt treatment on pregnancy characteristics. (A) Maternal body weight was recorded during mid—late pregnancy. Placental weight (B), litter size (C), and maternal net body weight (D) were recorded immediately after delivery (n = 10/group). Data are presented as mean±SEM. **P<0.01, ***P<0.001.

Maternal weight increased gradually during the gestational period in WT and KO mice given regular drinking water. Maternal weight was significantly lower in the experimental groups than in the control groups (Fig 4A). Among high salt-treated mice, body weight was significantly greater in the KO group than in the WT group (31.5 vs. 24.8 g, P<0.001; Fig 4A). Placental weight and litter size did not differ among all experimental groups (Fig 4B and 4C). Maternal net body weights of KO and WT mice given regular water were similar (29.3±2.7 and 27.9±2.7 g, respectively), whereas net body weight was greater in high salt-treated KO mice than in their WT counterparts (23.4±3.1 vs. 18.8±2.1 g, P<0.01; Fig 4D).

### High salt treatment increased blood perfusion and apoptosis resistance in the placentas of KO mice

**Fig 5:**
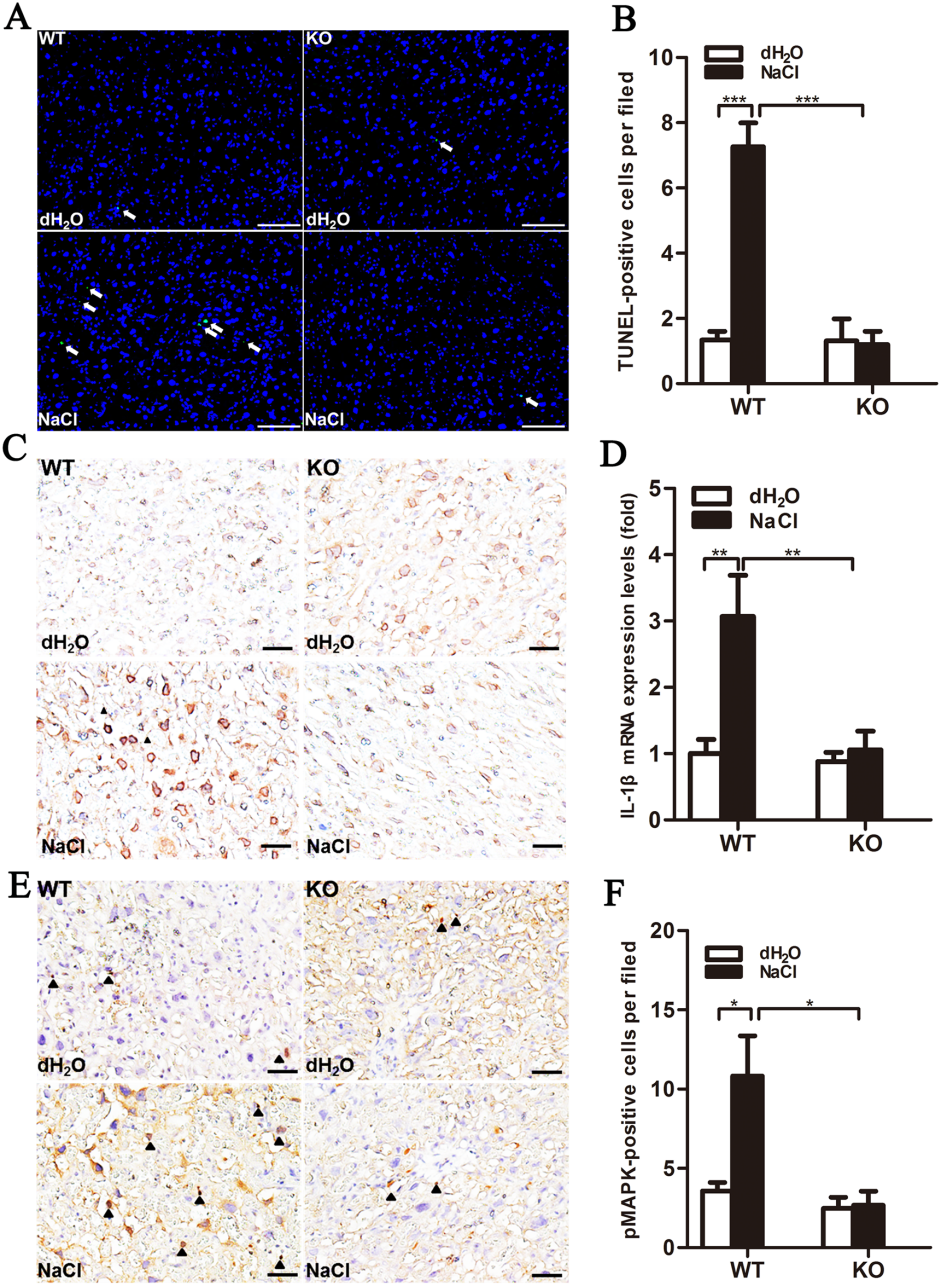
Increased numbers of apoptotic cells in placenta labyrinths of high salt-treated WT mice through IL-1β signaling pathway activation. (A) TUNEL-positive cells (green, arrows) were observed in placenta. DAPI (blue) was used for nuclear staining. Scale bar = 100 μm. (B) DAPI/TUNEL double-positive cells in placenta labyrinth were quantified in 10–15 fields/placenta and averaged. n = 4. Immunohistochemical imaging of IL-1β (C) and Il1b mRNA expression (D), detected by real-time qPCR (n = 5/group). (E) Immunohistochemical imaging of phosphorylated p38-MAPK (arrowhead show positive signals, hematoxylin co-stained with nucleus). Scale bar = 500 μm. (F) Positive cells were quantified in 10–15 fields/placenta and averaged (n = 4/group).

Renal and placental tissue sections showed no significant alteration in regular water- or high salt-treated WT or KO mice. However, increased blood flow with numerous red blood cells was found in the vascular sinuses of the placenta labyrinths of KO mice, indicating the presence of hemostatic defects (S3 Fig). Markedly increased numbers of apoptotic cells were identified as DAPI/TUNEL double positive in the placenta labyrinths of high salt-treated WT mice compared with KO mice (P<0.001; Fig 5A and 5B).

### High salt treatment induced the IL-1β signaling pathway in the placentas of WT mice

IL-1β protein and mRNA levels were upregulated in high salt-treated WT placentas (P<0.01; Fig 5C and 5D). The number of cells expressing phosphorylated p38-MAPK in placenta labyrinths was increased in high salt-treated WT mice, but not in their KO counterparts (P<0.05; Fig 5E and 5F). These data demonstrate that high salt drinking water upregulated TXAS expression and induced the IL-1β signaling pathway in the placenta, which may be related to the role of TXA2 in the regulation of IL-1β and its downstream molecules. These effects were minimal in TXAS-KO mice.

## Discussion

Poor placental development is accepted as a key factor in the etiology of PIH and PE.[3, 4] In this study, we demonstrated that TXAS mRNA and protein were highly expressed in placental tissue from patients with PE in comparison with gestational age—matched controls. This finding is in agreement with those of previous reports showing abundant TXAS mRNA and protein in the trophoblast layer, decidua, and blood vessels of placentas from PE pregnancies [37, 38]. It is also in concordance with the observation of elevated TXA_2_ metabolites in the circulation of pregnant women with PE [39], and suggests that placental TXA_2_ levels are correlated with the pathogenesis of PE.

Low-dose aspirin, which selectively inhibits TXA_2_ production, has shown beneficial effects in a wide range of clinical trials for the prevention of placenta-associated pregnancy complications, including 17–21% reduction of PE risk [40–45]. Recently, the US Preventive Services Task Force recommended the use of low-dose aspirin (81 mg daily) for women at high risk of developing PE [46, 47]. However, the mechanism underlying the protective effects of aspirin has not been well defined. In the present induced PE-like mouse model, established to address the role of TXA_2_ blockade in aspirin-mediated prevention of PE, pregnant WT mice demonstrated hypertension, imbalances in plasma prostanoid levels (elevated TXA_2_ level with no change in PGI_2_ level), increased TXAS in placenta, and decreased pup weight and size. These phenotypes are largely consistent with those observed in a previous rat model, established similarly with saltwater [29]. TXAS-KO mice, however, did not show such severe adverse effects; although maternal body weight increased less in the KO group, high blood pressure did not develop and pup weight and size decreased only slightly. The mechanism by which TXA_2_ affected maternal body weight, however, remains unclear.

The thromboxane receptor (TP) overexpression and synthetic TXA_2_ infusion have been shown to induce IUGR in pregnant rodents [48–50]. In addition, the inhibition of TXA_2_ production has been demonstrated to reduce PIH and proteinuria in a small-scale clinical trial involving women with PE. Building upon our previous demonstration of normal growth and thrombopoiesis, with only a mild hemostatic defect, in Tbxas1 KO mice [31], we demonstrated in the present study that TXA_2_ deletion did not result in reduced litter size, or a significant alteration in plasma sFlt-1 level. Together, our results confirm the protective effect of TXA_2_ blockade on the prevention of PIH, PE, and IUGR, and the safety of specific TXA_2_ inhibition.

Infusion with TXA_2_ analog in the last week of C57BL/6J mouse gestation was shown to induce IUGR [50]. Culturing of trophoblasts from clinical PE samples under hypoxia was found to induce TXAS expression [51], which may inhibit trophoblast differentiation and enhance apoptosis [15]. TXA_2_ is also considered to enhance oxidative stress, as it was shown to mediate superoxide production by neutrophils obtained from pregnant women [52]. All of this evidence suggests that TXA_2_ can damage placental tissues and cause placental insufficiency and IUGR [53]. Histological data from the present study suggest that TXA_2_ depletion increases blood infusion into the placenta, increasing nutrient supply to the fetus and thereby conveying resistance to high-salt drinking water—induced severe growth restriction.

Increased production of pro-inflammatory cytokines, such as IL-1β and IL-6, has been detected in women with PE [54]. IL-1β has been shown to modulate human trophoblast proliferation by inducing cell cycle arrest and triggering cellular apoptosis via p38-MAPK activation [55–58]. In the present study, *Il1b* mRNA was upregulated in placental tissue in WT, but not KO, mice treated with saltwater, accompanied by increased apoptosis. These data suggest that increased TXAS activates the IL-1β signaling pathway and induces apoptosis in PE placentas, which may lead to maternal hypertension [4]. Taken together, our findings suggest that TXA_2_ blockade inhibits IL-1β signaling pathway upregulation, thereby protecting placental cells from apoptosis and avoiding the development of maternal hypertension.

In summary, our study suggests that TXA_2_ plays an important role in hypertension development through the IL-1β signaling pathway in placenta, and also provide a potential explanation of the mechanism by which aspirin prevents high-risk PE. The study provides evidence supporting the safety of TXAS inhibitors, which may have anti-PE effects by preventing hypertension and placental damage. The findings thus support previous evidence that low-dose aspirin reduces PE risk in pregnant women [22].

## Supporting Information

S1 Fig Experimental design. Female mice were mated, plugs were checked on gestational day 0 (D0), and body weight was measured and mice were assigned randomly to groups on D10. Pregnant WT and KO mice were given dH_2_O and 2.7% NaCl in drinking water, respectively, from D12 until delivery. Maternal weight, blood pressure, and foot size were recorded every 2 days from D11 to D18. Blood pressure was measured and recorded using a tail-cuff system between 9:00 and 12:00. Urine was collected on D11, D14, and D18. Blood and tissue were collected immediately after delivery.
(TIF)

S2 Fig Maternal phenotypes of high salt treatment. Maternal blood pressure (A) SBP and (B) DBP were measured and recorded (baseline SBP = 99 mmHg, baseline DBP = 64 mmHg). (C) Maternal foot size was measured using electronic calipers. (D) Urine protein level was determined by protein assay. (E) Plasma sFlt-1 level was determined by EIA. Data are presented as mean±SEM. *P<0.05, **P<0.01.
(TIF)

S3 Fig Histological analysis of mouse placenta and kidney. Placenta (A) and kidney(B) samples were stained with H&E. (C) Glomeruli in kidney were quantified in 10–15 fields/placenta and averaged (n = 4/group). Data are presented as mean±SEM. Scale bar = 50 μm.
(TIF)
