# Supplementary figures and images for: Lack of Thromboxane Synthase Prevents Hypertension and Fetal Growth Restriction after High Salt Treatment during Pregnancy
Source: PLoS One. 2016 Mar 14;11(3):e0151617. doi: 10.1371/journal.pone.0151617 (PMC4790927; doi:10.1371/journal.pone.0151617)

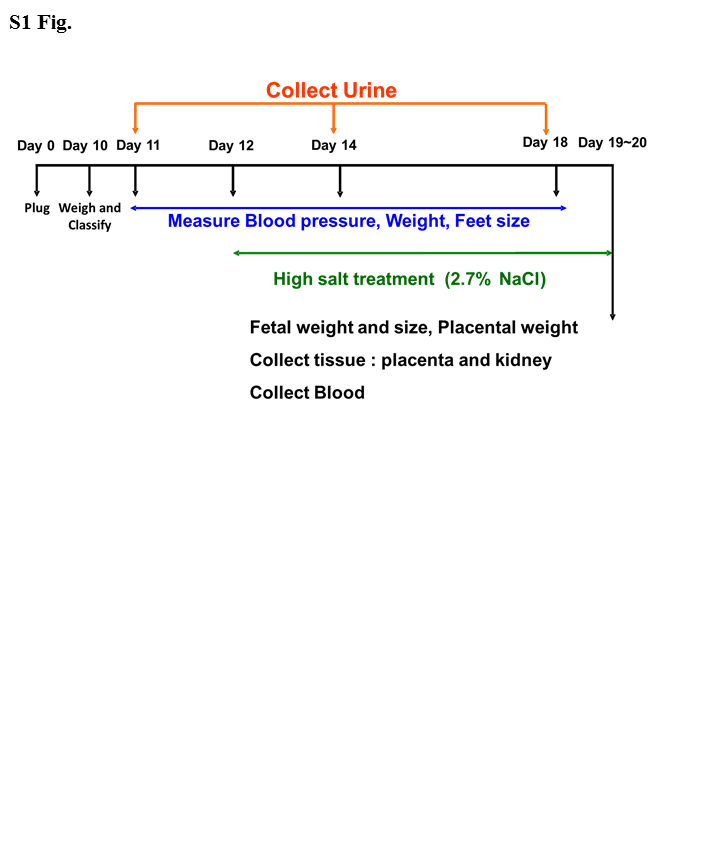

Supplement: S1 Fig — Female mice were mated, plugs were checked on gestational day 0 (D0), and body weight was measured and mice were assigned randomly to groups on D10. Pregnant WT and KO mice were given dH2O and 2.7% NaCl in drinking water, respectively, from D12 until delivery. Maternal weight, blood pressure, and foot size were recorded every 2 days from D11 to D18. Blood pressure was measured and recorded using a tail-cuff system between 9:00 and 12:00. Urine was collected on D11, D14, and D18. Blood and tissue were collected immediately after delivery. (TIF) [file pone.0151617.s001.tif]

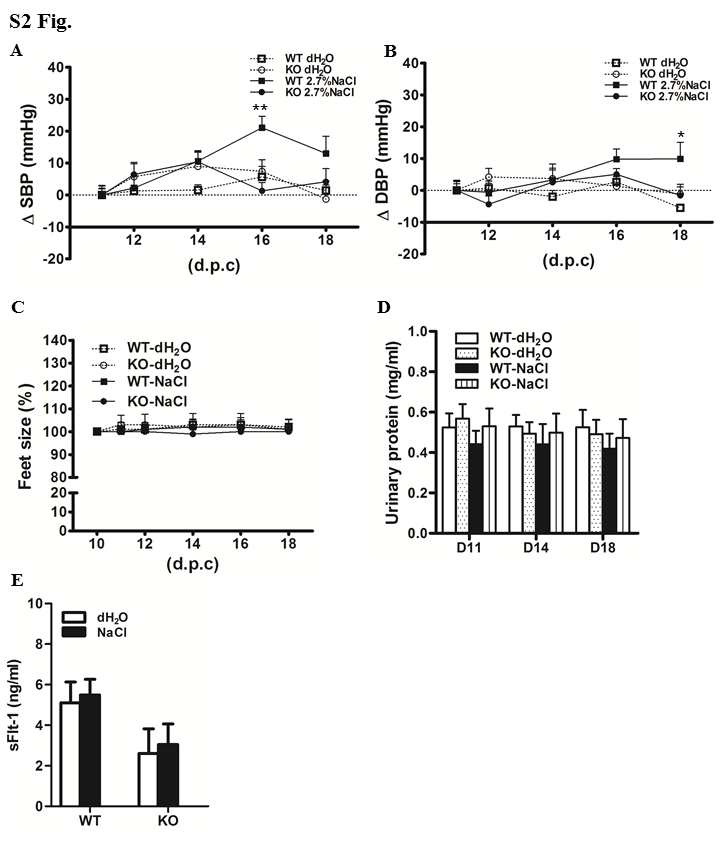

Supplement: S2 Fig — Maternal blood pressure (A) SBP and (B) DBP were measured and recorded (baseline SBP = 99 mmHg, baseline DBP = 64 mmHg). (C) Maternal foot size was measured using electronic calipers. (D) Urine protein level was determined by protein assay. (E) Plasma sFlt-1 level was determined by EIA. Data are presented as mean±SEM. *P<0.05, **P<0.01. (TIF) [file pone.0151617.s002.tif]

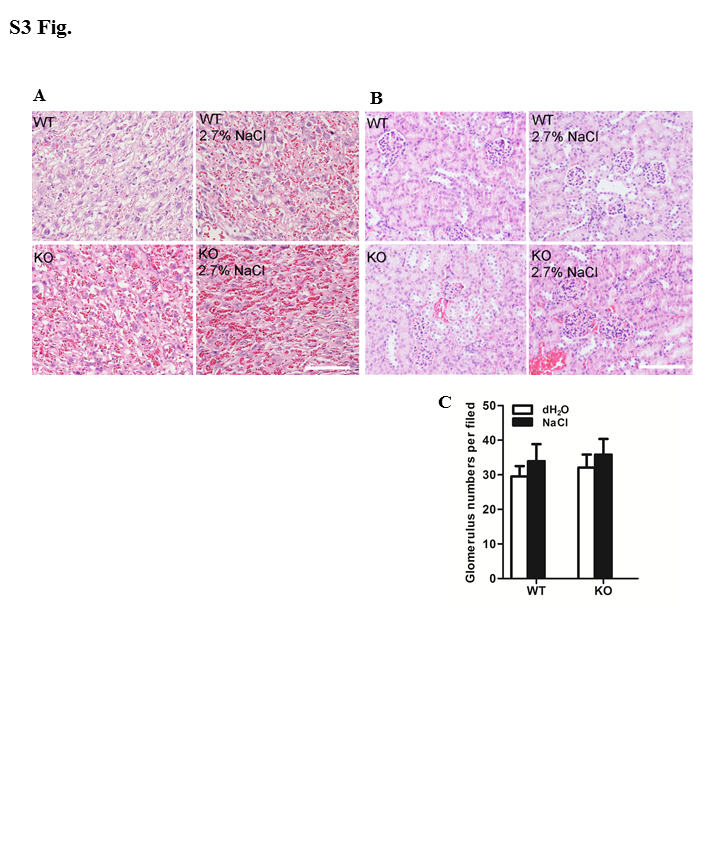

Supplement: S3 Fig — Placenta (A) and kidney(B) samples were stained with H&E. (C) Glomeruli in kidney were quantified in 10–15 fields/placenta and averaged (n = 4/group). Data are presented as mean±SEM. Scale bar = 50 μm. (TIF) [file pone.0151617.s003.tif]
